# Commentary: Microbiota-derived metabolites and cognitive dysfunction in dialysis patients: mechanisms and targeted therapeutic strategies

**DOI:** 10.3389/fmicb.2026.1948248

**Published:** 2026-09-16

**Authors:** Shengchao Zhang, Tong Li, Yong Liu

**Affiliations:** 1 Department of Rehabilitation Medicine, The First Affiliated Hospital of Dalian Medical University, Dalian, China; 2 College of Health-Preservation and Wellness, Dalian Medical University, Dalian, China

**Keywords:** cognitive impairment, dialysis, gut microbiota, metabolite kinetics, protein-bound uremic toxins

## 1 Introduction

We read with great interest the review by Xiao et al. (2026), which integrates gut dysbiosis, microbial metabolism, uremic toxicity, blood–brain barrier injury, and cognitive dysfunction in dialysis patients. The authors appropriately acknowledge that direct evidence in dialysis populations remains limited and that many proposed mechanisms are inferred from observational studies, experimental models, or non-dialysis chronic kidney disease.

The next step may therefore be not simply to identify further associations between bacterial taxa, plasma metabolites, and cognitive scores, but to determine whether the microbiota makes a measurable contribution to systemic metabolite exposure and whether modifying that contribution improves neurological outcomes. This is particularly challenging in dialysis patients, because circulating metabolite levels reflect intestinal production, host biotransformation, protein binding, residual kidney function, and dialysis-dependent removal.

## 2 From circulating concentration to attributable exposure

Indoxyl sulfate and p-cresyl sulfate illustrate this problem. Their plasma concentrations arise from intestinal precursor production, host conjugation, renal tubular secretion, and removal through dialysis or residual kidney function. Clearance studies have shown that both intestinal generation and renal elimination contribute to systemic concentrations, with tubular secretion playing an important role (Poesen et al., 2013). In hemodialysis patients, free and total concentrations are not interchangeable, because albumin binding influences the biologically available fraction (Meijers et al., 2009). Protein-bound uremic toxin levels are also closely associated with residual kidney function and may not be substantially reduced by convective dialysis alone (van Gelder et al., 2020).

An identical fall in plasma concentration may therefore represent different biological events. A prebiotic may reduce microbial precursor generation; an oral adsorbent may limit intestinal absorption; preservation of residual kidney function may enhance endogenous elimination; and an extracorporeal adsorptive technique may increase blood removal. Although each intervention could lower the measured toxin level, only some would demonstrate that microbiota modulation was responsible.

We suggest interpreting metabolite exposure through a production–transformation–binding–clearance framework. Trials of microbiota-directed therapies should assess microbial functional activity or precursor production rather than relying solely on taxonomic abundance. Measurements should also include free and total toxin concentrations, serum albumin, residual urine output or clearance, dietary substrate intake, and dialytic solute removal. Otherwise, it remains difficult to determine whether a lower plasma concentration reflects reduced microbial generation, altered host handling, or improved clearance.

## 3 Dialysis modality and measurement timing belong within the causal model

Hemodialysis and peritoneal dialysis should not be treated only as background characteristics. They create distinct exposure dynamics. Hemodialysis produces intermittent changes in solute concentrations, fluid balance, blood pressure, and cerebral perfusion. Peritoneal dialysis provides more continuous clearance, but its effects vary with residual kidney function, peritoneal transport status, dialysis prescription, and glucose exposure.

These differences also affect cognitive assessment. Hemodialysis can acutely reduce cerebral blood flow, suggesting that the procedure itself may influence neurological function independently of microbial metabolites (Polinder-Bos et al., 2018). Cognitive performance may also vary depending on whether testing occurs before, during, or after dialysis and on the testing environment (Tholen et al., 2014). A score obtained immediately after treatment may therefore reflect fatigue, hemodynamic stress, or acute cerebral changes in addition to longer-term toxin exposure.

If stool, blood, and cognitive data are collected at inconsistent time points, transient dialysis-related effects may be mistaken for stable microbiota–metabolite associations. Hemodialysis studies should prespecify sampling and cognitive-testing windows. Peritoneal dialysis studies should document exchange timing, prescription, residual kidney function, recent peritonitis, and antibiotic exposure. These factors influence both metabolite kinetics and cognition and should be incorporated into the causal model rather than treated merely as confounders.

## 4 From biomarker change to cognitive mediation

The interventions reviewed by Xiao et al. (2026) include dietary modification, probiotics, prebiotics, synbiotics, fecal microbiota transplantation, intestinal adsorbents, and enhanced toxin removal. Most available studies, however, report changes in microbial composition, circulating metabolites, inflammation, or kidney-related outcomes. Direct evidence that these interventions improve cognition through a microbiota-dependent mechanism remains scarce.

A reduction in a circulating toxin should therefore be viewed as an intermediate biological response, not as a validated surrogate for cognitive benefit. A more informative trial would examine the following sequence:

Intervention → microbial functional change → altered precursor or metabolite exposure → reduced neurovascular or inflammatory injury → cognitive benefit.

Each step requires specific evidence. Microbial target engagement could be assessed through metagenomic or metatranscriptomic pathways and precursor measurements. Systemic target engagement would require free and total metabolite concentrations together with clearance data. Neurological effects could be evaluated using blood–brain barrier markers, inflammatory biomarkers, cerebral perfusion, or neuroimaging, while clinical benefit should be assessed with repeated, domain-specific cognitive tests. Mediation analysis could then determine whether changes in metabolite exposure account for any observed cognitive improvement.

## 5 Discussion

The microbiota–gut–brain–kidney axis described by Xiao et al. provides a useful framework for cognitive dysfunction in dialysis patients. Its translational value will depend on whether microbial metabolism can be separated from other determinants of systemic exposure.

The central question is not merely whether a metabolite is associated with the microbiota or cognition, but whether microbial production contributes measurably to that exposure, whether an intervention modifies this component, and whether the resulting change improves neurological and cognitive outcomes. Combining microbial functional measurements with toxin kinetics, dialysis characteristics, neurological biomarkers, and standardized cognitive assessments would turn this associative framework into a testable causal model.
